# The complete chloroplast genome sequence of *Artemisia ordosica*

**DOI:** 10.1080/23802359.2020.1768946

**Published:** 2020-05-27

**Authors:** Xiang Li, Jinhuan He, Yanxian Lian, Fengling Li, Jianghua Suo, Guoqian Jin

**Affiliations:** a College of Food and Bioengineering, Henan University of Animal Husbandry and Economy, Zhengzhou, China; b School of Traditional Chinese Pharmacy, China Pharmaceutical University, Nanjing, China

**Keywords:** *A. ordosica*, chloroplast genome, phylogenetic analysis, genetic information

## Abstract

The complete chloroplast genome sequence of *Artemisia ordosica* was characterized from Illumina pair-end sequencing. The chloroplast genome of *A. ordosica* was 151,209 bp in length, containing a large single-copy region (LSC) of 80,975 bp, a small single-copy region (SSC) of 16,002 bp, and two inverted repeat (IR) regions of 27,116 bp. The overall GC content is 30.71%, while the correponding values of the LSC, SSC, and IR regions are 64.2%, 69.3%, and 60.0%, respectively. The genome contains 138 complete genes, including 91 protein-coding genes (62 protein-coding gene species), 39 tRNA genes (29 tRNA species) and 8 rRNA genes (4 rRNA species). The Neighbour-joining phylogenetic analysis showed that *A. ordosica* and *Artemisia scoparia* clustered together as sisters to other *Artemisia* species.

## Introduction

Animal diets supplemented with natural herbs or plant-derived products rich in phenols and flavonoids can relieve intestinal damage caused by stress. Artemisia plants contain many active ingredients, including flavonoids, flavonoids, polysaccharides, sterols and polyacetylene. Some products of this genus have been shown to have anti-malarial, anti-viral, anti-tumor, antipyretic, hemostatic, anticoagulant, antianginal, antioxidant, anti-hepatitis, anti-ulcer and anti-spasm capabilities, and have good development in animal husbandry prospects (Neale and Antoine 2011). *Artemisia ordosica* is a perennial herb as a member of the Artemisia belonged to the family Asteraceae and widely distributed in China, which has persisted largely in an undomesticated state that is highly resistant to different environmental stresses (Hou et al. 2018). Moreover, we can develop conservation strategies easily when we understand the genetic information of *A. ordosica*. In the present research, we constructed the whole chloroplast genome of *A. ordosica* and understood many genome varition information about the species, which will provide beneficial help for population genetics studies of *A. ordosica.*

The fresh leaves of *A. ordosica* were collected from Yulin (109°44 ′E; 38°17′N). Fresh leaves were silica-dried and taken to the laboratory until DNA extraction. The voucher specimen (YH001) was laid in the Herbarium of Nanyang Institute of Technology and the extracted DNA was stored in the −80 °C refrigerator of the Key Laboratory of School of Biological and Chemical Engineering. We extracted total genomic DNA from 25 mg silica-gel-dried leaf using a modified CTAB method (Doyle 1987). The whole-genome sequencing was then conducted by Biodata Biotechnologies Inc. (Hefei, China) with Illumina Hiseq platform. The Illumina HiSeq 2000 platform (Illumina, San Diego, CA) was used to perform the genome sequence. We used the software MITObim 1.8 (Hahn et al. 2013) and metaSPAdes (Nurk et al. 2017) to assemble chloroplast genomes. We used *Artemisia* species (GenBank: NC045286) as a reference genome. We annotated the chloroplast genome with the software DOGMA (Wyman et al. 2004), and then corrected the results using Geneious 8.0.2 (Campos et al. 2016) and Sequin 15.50 (http://www.ncbi.nlm.nih.gov/Sequin/).

The complete chloroplast genome of *A. ordosica* (GenBank accession number NC046571) was characterized from Illumina pair-end sequencing. The complete chloroplast genome sequence of *A. ordosica* was characterized from Illumina pair-end sequencing. The chloroplast genome of *A. ordosica* was 151,209 bp in length, containing a large single-copy region (LSC) of 80,975 bp, a small single-copy region (SSC) of 16,002 bp, and two inverted repeat (IR) regions of 27,116 bp. The overall GC content is 30.71%, while the correponding values of the LSC, SSC, and IR regions are 64.2%, 69.3%, and 60.0%, respectively. The genome contains 138 complete genes, including 91 protein-coding genes (62 protein-coding gene species), 39 tRNA genes (29 tRNA species) and 8 rRNA genes (4 rRNA species).

**Figure 1.**
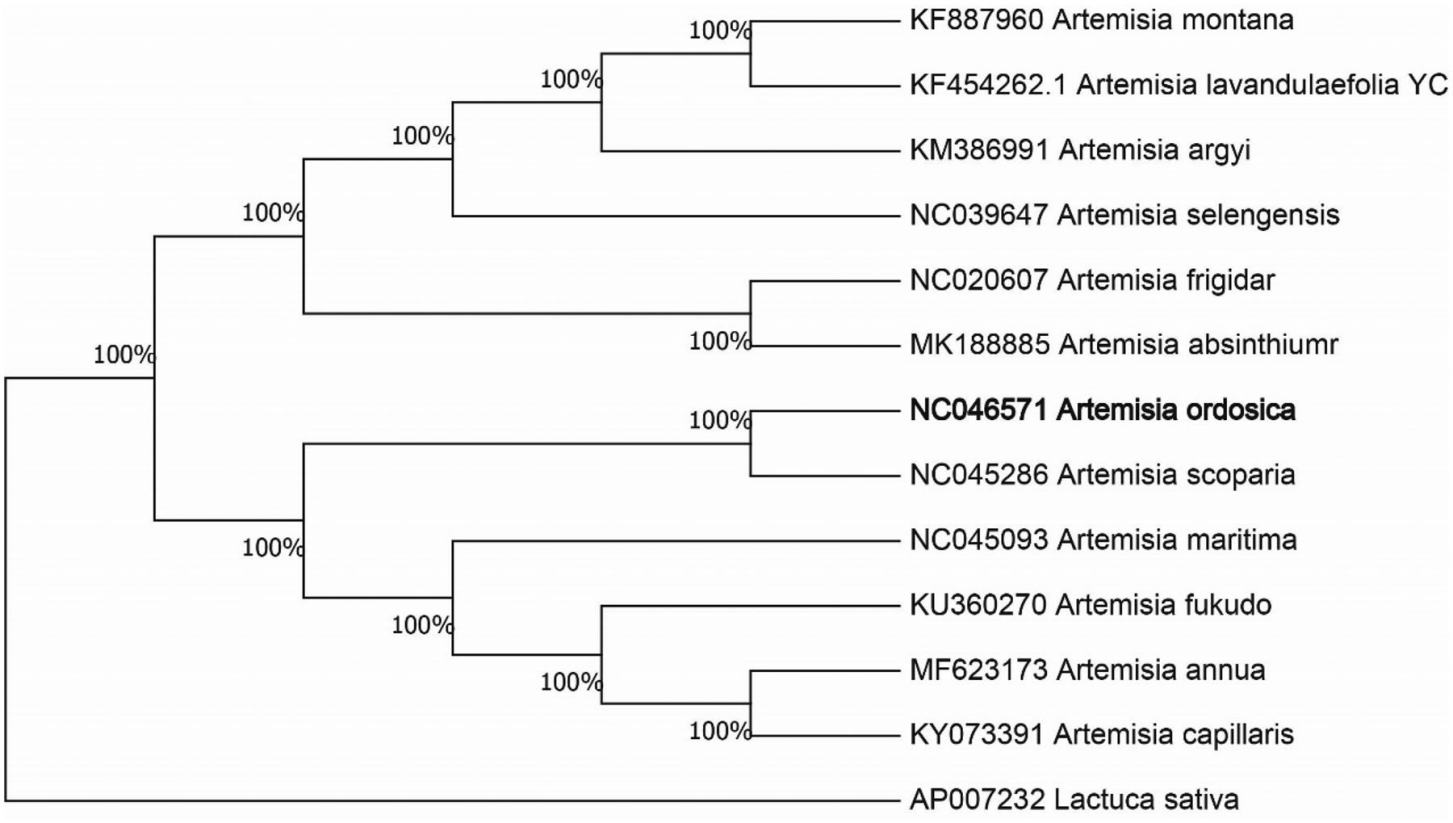
Neighbour-joining (NJ) analysis of *A. ordosica* and other related species based on the complete chloroplast genome sequence. *Lactuca sativa* (AP007232) was set as the outgroup. All other sequences were downloaded from NCBI GenBank.

We used the complete chloroplast genomes sequence of *A. ordosica* and 11 other related species of *Artemisia* and *Lactuca* sativa as outgroup to construct phylogenetic tree. The 13 chloroplast genome sequences were aligned with MAFFT (Katoh and Standley 2013), and then the Neighbour-joining tree was constructed by MEGA 7.0 (Kumar et al. 2016). The results confirmed that *A. ordosica* was clustered with *A. scoparia* (Figure 1).

## Data Availability

The data that support the findings of this study are openly available in GenBank at https://www.ncbi.nlm.nih.gov/nuccore/NC_046571.1, reference number NC046571.
